# 835. Histoplasmosis in Western India: Shedding Light on a Potentially Underdiagnosed Disease – A Prospective Study from a Tertiary care centre

**DOI:** 10.1093/ofid/ofad500.880

**Published:** 2023-11-27

**Authors:** Akshatha Ravindra, Deepak Kumar, Santhanam Naguthevar, Gopal Krishana Bohra, Yash Khatod, Neetha T R, Shivang Sharma, Navneet Kaur, Durga Shankar Meena, Naresh Kumar Midha, M K Garg

**Affiliations:** AIIMS Jodhpur, Jodhpur, Rajasthan, India; All India Institute of Medical Sciences, Jodhpur (India), Jodhpur, Rajasthan, India; AIIMS JODHPUR, Jodhpur, Rajasthan, India; AIIMS Jodhpur, Jodhpur, Rajasthan, India; AIIMS Jodhpur, Jodhpur, Rajasthan, India; AIIMS Jodhpur, Jodhpur, Rajasthan, India; AIIMS, Jodhpur, Rajasthan, India; AIIMS Jodhpur, Jodhpur, Rajasthan, India; AIIMS, Jodhpur, Rajasthan, India; AIIMS, Jodhpur, Rajasthan, India; AIIMS, Jodhpur, Rajasthan, India

## Abstract

**Background:**

Histoplasmosis is a fungal infection caused by Histoplasma capsulatum, can range from pulmonary to disseminated infections. While it is endemic in certain regions of the world, including North and South America, Africa, and parts of Asia, it is considered rare in India. However, recent reports suggest an increasing incidence of histoplasmosis in India. This project aims to evaluate the prevalence of histoplasmosis in the non-endemic western part of India and to study its clinical profile.

**Methods:**

This was a prospective cross-sectional study which was conducted between January 2022 – February 2023 in Medicine department, AIIMS Jodhpur after obtaining approval from Ethics Committee. Patients aged more than 18 years with clinically suspected disseminated histoplasmosis (PUO, pancytopenia etc.) was screened using a rapid lateral flow test – OIDx Histoplasma Capsulatum LFA Kit. Urine samples were collected from all participants. Additional samples such as Blood, cerebrospinal fluid (CSF), biopsies etc, were collected for further confirmatory tests from patients with positive urine Histoplasma test as part of routine clinical care.

**Results:**

A total of 45 patients (29 males, 64.4%) with a mean age of 48.8 years (range, 20-78) were included. Urinary histoplasma was found to be positive in 15 patients (33.33%), 8 of whom were confirmed with histopathology. The most common risk factor among the patients was HIV (35.5%). The common symptoms observed were fever and weight loss (73.3%), followed by cough (53.3%). Disseminated histoplasmosis with hepatosplenomegaly was seen in nearly 46.6% of the cases, while adrenal involvement was seen in 33.3%. Skin lesions were observed in only one-third of the patients. All patients were treated with Inj Amphotericin B, followed by itraconazole based on their weight. Most patients responded well to the treatment, with only one mortality.

**Table 1**

**Figure:**
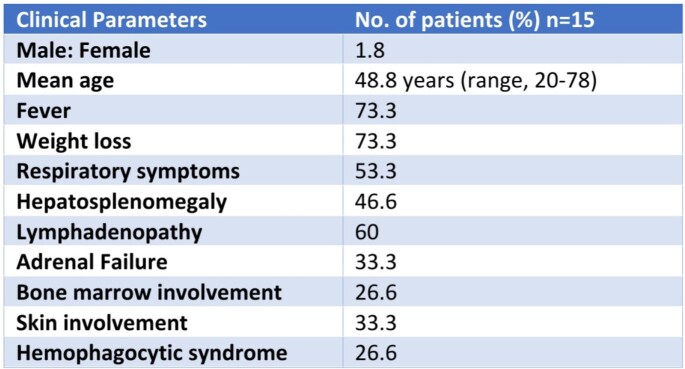

Clinical features of Disseminated Histoplasmosis in our study

**Figure:**
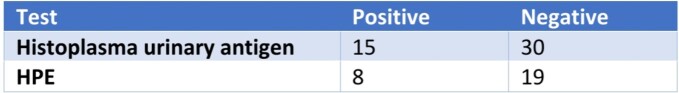

**Conclusion:**

The prevalence of histoplasmosis was found to be significant, particularly among HIV-positive patients. These findings emphasize the importance of considering histoplasmosis in the differential diagnosis of patients with compatible symptoms, especially in HIV-positive patients, and the need for further studies to determine the true burden of this disease in India.

**Disclosures:**

**All Authors**: No reported disclosures

